# Benzylpenicillin versus wide-spectrum beta-lactam antibiotics as empirical treatment of *Haemophilus influenzae*-associated lower respiratory tract infections in adults; a retrospective propensity score-matched study

**DOI:** 10.1007/s10096-018-3311-x

**Published:** 2018-06-30

**Authors:** John Thegerström, Viktor Månsson, Kristian Riesbeck, Fredrik Resman

**Affiliations:** 1 Riesbeck laboratory, Clinical Microbiology, Department of Translational Medicine, Faculty of Medicine, Lund University, Jan Waldenströms gata 59, SE-205 02 Malmö, Sweden; 2 Infectious Diseases Research Unit, Department of Translational Medicine, Faculty of Medicine, Lund University, Ruth Lundskogs gata 3, SE-205 02 Malmö, Sweden

**Keywords:** *Haemophilus influenzae*, Benzylpenicillin, Beta-lactam antibiotics, Community-acquired pneumonia, Empirical antibiotic treatment, Propensity score

## Abstract

There is consensus that definitive therapy for infections with *H. influenzae* should include antimicrobial agents with clinical breakpoints against the bacterium. In Scandinavia, benzylpenicillin is the recommended empirical treatment for community-acquired pneumonia (CAP) except in very severe cases. However, the effect of benzylpenicillin on *H. influenzae* infections has been debated. The aim of this study was to compare the outcomes of patients given benzylpenicillin with patients given wide-spectrum beta-lactams (WSBL) as empirical treatment of lower respiratory tract *H. influenzae* infections requiring hospital care. We identified 481 adults hospitalized with lower respiratory tract infection by *H. influenzae*, bacteremic and non-bacteremic. Overall, 30-day mortality was 9% (42/481). Thirty-day mortality, 30-day readmission rates, and early clinical response rates were compared in patients receiving benzylpenicillin (*n* = 199) and a WSBL (*n* = 213) as empirical monotherapy. After adjusting for potential confounders, empirical benzylpenicillin treatment was not associated with higher 30-day mortality neither in a multivariate logistic regression (aOR 2.03 for WSBL compared to benzylpenicillin, 95% CI 0.91–4.50, *p* = 0.082), nor in a propensity score-matched analysis (aOR 2.14, 95% CI 0.93–4.92, *p* = 0.075). Readmission rates did not significantly differ between the study groups, but early clinical response rates were significantly higher in the WSBL group (aOR 2.28, 95% CI 1.21–4.31, *p* = 0.011), albeit still high in both groups (84 vs 81%). In conclusion, despite early clinical response rates being slightly lower for benzylpenicillin compared to WSBL, we found no support for increased mortality or readmission rates in patients empirically treated with benzylpenicillin for lower respiratory tract infections by *H. influenzae*.

## Introduction

*Haemophilus influenzae* is considered the second most common bacterial cause of community-acquired pneumonia (CAP) after *Streptococcus pneumoniae* [1]. Surveillance data have suggested an increased incidence of invasive infections with *H. influenzae* in recent years, and since the introduction of capsule type b polysaccharide conjugate vaccines, non-encapsulated strains (NTHi) dominate, followed by capsule type f [2–4]. This has led to a shift in the clinical epidemiology of severe *H. influenzae* infections, as most cases now present as pneumonia in older adults or patients with comorbidities, most notably chronic obstructive pulmonary disease (COPD) [2, 3, 5]. With the widespread introduction of conjugated pneumococcal vaccines, there is also concern that the proportion of CAP caused by *H. influenzae* may increase [6]. Although this has yet to be confirmed, one recent study from Great Britain using molecular diagnostics showed it to be the most common agent in CAP, contributing to 40% of cases [7].

In Scandinavian countries, high-dose benzylpenicillin (PcG) is still recommended as the empirical treatment for patients with CAP requiring hospitalization, with the exception of patients with immunosuppression or very severe presentation (CRB-65 > 2 or concomitant severe sepsis [8]). There is a long tradition of benzylpenicillin treatment in Scandinavia, which remains effective against the majority of pneumococci and has limited collateral ecological effects compared to other empirical alternatives [9].

The activity of benzylpenicillin against *H. influenzae* has been debated. Recommendations are based mainly on clinical experience combined with theoretical assumptions from PK/PD simulations and time-killing experiments [10]. The European Committee on Antimicrobial Susceptibility Testing (EUCAST) has not defined clinical breakpoints for benzylpenicillin, referring to insufficient evidence from clinical studies [11]. Moreover, in recent years, the proportion of *H. influenzae* isolates with reduced susceptibility to aminopenicillins due to alterations in penicillin-binding protein 3 (termed rPBP3) has increased, reaching 10–20% [12, 13]. In addition, a stable proportion (an additional 10–20%) of isolates expresses beta-lactamases [14, 15]. Both of these resistance mechanisms are likely to increase the risk of treatment failure with use of empirical benzylpenicillin. The increase of these resistance mechanisms may make benzylpenicillin a less viable option in the case of *H. influenzae* CAP. One recent retrospective study from Denmark showed a significantly higher 30-day mortality for patients receiving benzylpenicillin as definitive treatment for *H. influenzae* bacteremia, compared to those who received cephalosporins or aminopenicillins [16].

Considering the proposed increase in the proportion of *H. influenzae* as a cause of CAP and the current empirical treatment recommendation of benzylpenicillin, there is a need to assess the outcome of empirical benzylpenicillin treatment in cases of lower respiratory tract infection by *H. influenzae*. The primary objective of this study was to compare the 30-day mortality between adults who had received benzylpenicillin as empirical treatment for *H. influenzae* lower respiratory tract infections (with or without bacteremia) requiring hospital care with individuals who had received empirical treatment with wide-spectrum beta-lactams (WSBL). As secondary objectives, 30-day readmission rates and estimations of early clinical response were compared between the two groups.

## Materials and methods

### Study population and setting

Patients with positive cultures of *H. influenzae* were identified at the clinical microbiology laboratories in Malmö and Lund, Sweden. The catchment area of these laboratories corresponds to Skåne county in southern Sweden (adult population of 1,045,792 in 2016 [17]), and healthcare was provided by Skåne University Hospital and surrounding regional hospitals. All blood and respiratory tract cultures sampled in the catchment area were analyzed in these two laboratories.

### Case definitions and exclusion criteria

Two case definitions were applied: (1) bacteremia with *H. influenzae* in an individual ≥ 18 years of age 1997–2016 with a lower respiratory tract infection or (2) pure culture of *H. influenzae* from a respiratory tract sample in an individual hospitalized due to a lower respiratory tract infection 2015–2016.

Patients aged ≥ 18 years with positive blood cultures of *H. influenzae* between 1997 and 2016 were identified through the laboratories’ records. All individuals with positive blood cultures were included in the study if they had a concurrent respiratory tract infection, except for those with epiglottitis (in which the recommended treatment in the area is cefotaxim). Recurrent episodes of bacteremia were only recorded once.

Individuals above 18 years of age with positive cultures from sputum or nasopharynx in Skåne county in 2015–2016 were also identified. All individuals with positive cultures were included in the study if (1) they were admitted more than 24 h to a hospital ward due to a lower respiratory tract infection, (2) no other respiratory tract pathogen (see below) was present in any microbiological sample, (3) they had been diagnosed with lower respiratory tract infection (pneumonia or COPD exacerbation) at discharge from hospital, and (4) no alternative foci of infection were identified except the lower respiratory tract.

In cultures from sputum and nasopharyngeal swabs, concurrent growth of *Streptococcus pneumoniae*, beta-haemolytic streptococci and *Moraxella catharrhalis* were regularly sought for, and if any of these pathogens were identified in a culture, the patient was excluded. If a respiratory tract sample indicated the presence of influenza virus, respiratory syncytial virus, *Mycoplasma pneumoniae*, *Chlamydophila pneumoniae*, *Chlamydia psittaci*, *Legionella pneumophila*, or *Pneumocystis jirovecii* through polymerase chain reaction (PCR), the patient was excluded. Finally, patients were excluded if the presence of *Streptococcus pneumoniae* or *Legionella pneumophila* was demonstrated by urine antigen detection tests.

### Microbiological methods and antimicrobial susceptibility testing

All blood samples were cultured using the automated BacTAlert system (bioMérieux, Marcy l’Etoile, France) (1997–December 2014) or the BACTEC system (BD diagnostic systems, Sparks, MD) (December 2014–2016). Respiratory tract specimens from sputum and nasopharyngeal swabs were cultured using standard microbiological techniques. Isolates were identified by typical colony morphology on agar plates, through standard biochemical tests and by MALDI Biotyper analysis (Bruker Daltonics, Bremen, Germany). Capsule typing was performed by PCR for *H. influenzae* isolated from blood. Isolates from respiratory tract samples were not routinely capsule typed and were not saved (and thus not available for capsule typing at the time of the study).

Antimicrobial susceptibility testing was performed according to local laboratory guidelines until 2009, and thereafter according to the EUCAST algorithm [18], which is based upon a disk diffusion screen of 1 U benzylpenicillin on fastidious Mueller Hinton solid medium (MH-F) for identification of beta-lactam resistance. Beta-lactamase production was confirmed by a standard nitrocefin assay in screening-positive cases. Gradient tests (Etest, bioMérieux, Marcy l’Etoile, France) were used to determine the actual MICs of beta-lactam agents if the first disk diffusion screen was positive. Data on antimicrobial susceptibility, including beta-lactam resistance by rPBP3 and beta-lactamase production, were obtained from laboratory records.

### Clinical definitions

The following clinical descriptive patient data were recorded from hospital medical records: age, sex, immunosuppression, and comorbidities according to Charlson/Deyo comorbidity index [19]. The study definition of immunosuppression was ongoing primary immune deficiency or immunosuppressive therapy not denoted in the Charlson/Deyo comorbidity index, in order to avoid overfitting in the statistical analysis. Regarding the presenting infection, the following was recorded in addition to the antibiotic treatment strategy: sepsis severity score (SCCM/ESICM/ACCP/ATS/SIS criteria [20]), the maximal concentration of C-reactive protein during hospitalization, the CRB-65 score, and admittance to an intensive care unit. The following was recorded regarding the outcome of the infection: all-cause 30-day mortality, readmission to hospital within 30 days from discharge, and early clinical response. Early clinical response was defined as no signs of fever, tachycardia, hypotension, hypoxemia, or tachypnea on day 4 following admission, according to FDA criteria [21]. Since complete data on all parameters by day 4 were not always available, they were complemented by an evaluation of whether a substantial general improvement of the patient’s condition had occurred by day 4.

Patients were sorted into three groups according to type of empirical antibiotic therapy: (1) patients receiving intravenous (i.v.) benzylpenicillin as empirical therapy, (2) patients receiving any other i.v. beta-lactam agent with clinical breakpoints against *H. influenzae*, and (3) patients receiving other empirical treatment regimens. Empirical treatment was defined as the initial antibiotic agent the patient received upon admittance, prior to culture results. Clinical outcomes were compared between groups 1 and 2. In the multivariate regressions and propensity score-matched analyses, all patients who received concomitant empirical therapy with another antibiotic active against *H. influenzae* (fluoroquinolone, tetracycline, aminoglycoside) were excluded.

### Statistical analysis

Data were analyzed using Stata 14 (StataCorp, College Station, TX) and SPSS statistics version 24 (IBM, Armonk, NY). The results were expressed as counts and percentages for categorical variables and as medians and interquartile ranges for continuous variables. Comparisons of baseline statistics between the empirical treatment groups were assessed using Chi^2^-test for categorical variables, the Mann-Whitney *U* test or the Kruskal-Wallis test for continuous variables. *P* values ≤ 0.05 were considered statistically significant. Univariate logistic regressions were performed to establish associations between outcomes (30-day all-cause mortality, 30-day readmission, and early clinical response) and collected predictors as well as covariates.

Multivariate logistic regression models were fitted for the defined outcomes. The main predictor was empirical antibiotic treatment (benzylpenicillin monotherapy versus i.v. WSBL monotherapy). Considering the relatively low number of outcomes, to avoid over-fitting, all multivariate models were fitted using the purposeful selection algorithm [22]. Briefly, the main predictor and all covariates with a *p* value of < 0.2 in the univariate analysis were included in a crude model. The least significant covariate was step-wise removed from the model, unless the removal changed the adjusted odds ratios by more than 20%, until only significant and strongly influential covariates remained. A separate analysis was performed for 30-day mortality on only bacteremic patients.

A propensity score-matched analysis was performed to assess the effect of the two empirical treatment groups on 30-day mortality, 30-day readmission, and early clinical response. Propensity scores were calculated in a logistic regression using the treatment group as outcome. The following variables were used as covariates in this regression: age (categorized), sex, ICU-care, maximum CRP, CCI, bacteremia, immune suppression, and sepsis severity score. A 1–1 nearest neighbor matching without replacement was performed using the psmatch2 module [23], with a caliper of 0.2. The propensity scores were plotted graphically to verify spread and overlap, and balance in the covariates in the matched cohort was verified (Table 5). Finally, the associations between treatment and outcomes in the matched cohort were assessed in two ways, with full-cohort logistic regression as well as conditional logistic regression on matched pairs. In all analyses, assessments were made only on individuals with complete outcome data for the respective outcome.

The potential effect modification of beta-lactamase production and rPBP3 on the associations between treatment group and outcomes was investigated by stratifying the outcomes per treatment group and resistance mechanism in the propensity-matched cohort and comparing the odds ratios within each strata, including estimation of the beta-coefficient, 95% confidence interval, and *p* value for the respective interaction term in logistic regressions*.*

## Results

**Fig. 1:**
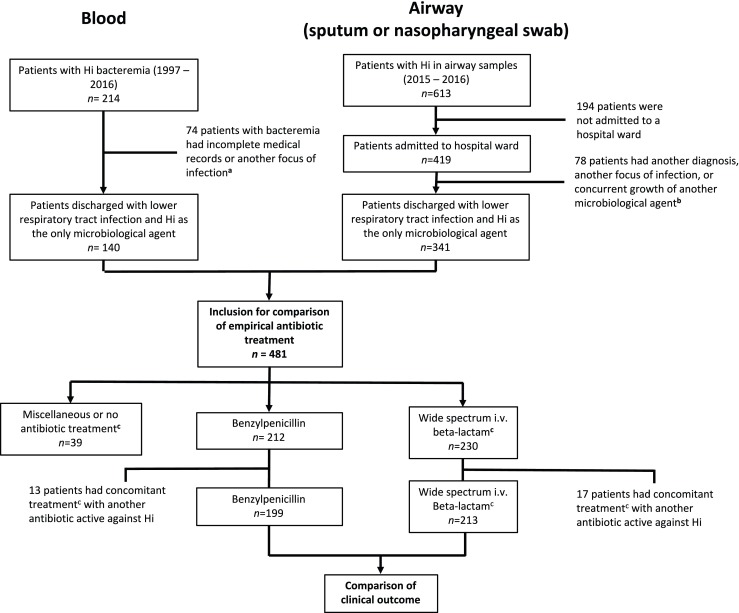
Cases included in the study. Flowchart summarizing the number of included and excluded patients in the study as well as the reason for exclusion. Hi = *Haemophilus influenzae*. ^a, b^See appendix Table 6 for diagnosis of the excluded patients. ^c^See appendix Table 7 for the specific antibiotic agents administered in the different groups

A total of 214 unique episodes of bacteremia with *H. influenzae* were identified in the catchment area between 1997 and 2016. Of these, 140 individuals had a lower respiratory tract infection (the vast majority had CAP, *n* = 135, 96%). In addition, a total of 613 unique adult patients had growth of *H. influenzae* in sputum or nasopharyngeal swabs taken at an emergency department in the catchment area between 2015 and 2016. Of these, 419 were admitted to a hospital ward for more than 24 h. A total of 341 of these patients had been diagnosed with a lower respiratory tract infection (the vast majority were CAP), while at the same time not meeting any of the defined microbiological exclusion criteria. Thus, in total 481 individual cases were included for further analysis (Fig. 1; Appendix Table 6).

### Descriptive and demographic characteristics of the crude study population

Out of the 481 included patients, 281 (58%) were women. The median age was 75 years (interquartile range (IQR), 66–84). A total of 25 patients (5.2%) met the study criteria of immunosuppression, while the median Charlson comorbidity index (CCI), unadjusted for age, was 2 (IQR 1–3). One out of four patients in the study (*n* = 120) had COPD. The most commonly confirmed diagnosis was pneumonia (*n* = 418, 87%) followed by COPD exacerbation (*n* = 39, 8%). A total of 55 patients (12%) had severe sepsis or septic shock upon admission, whereas 30 (6%) had a CRB-65 score of > 2. The median CRP level was 237 mg/L (IQR 157–318 mg/L), and 21 individuals (4%) were admitted to an ICU. In total, 108 isolates (23%) were screening positive for beta-lactam resistance through rPBP3 in screening, and 65 (14%) were beta-lactamase positive.

Forty-two individuals died within 30 days after cultures were being taken, resulting in an all-cause 30-day mortality of 9%. The 30-day mortality in patients with concurrent bacteremia was 12% (*n* = 17) and among patients with growth of *H. influenzae* exclusively in respiratory tract samples 7% (*n* = 25). The readmission rate 30 days after discharge was 15% (*n* = 72) for the whole study cohort, whereas 77 patients (16%) failed to meet the criteria of early clinical response on day 4.

Patients were sorted into three groups depending on which empiric antibiotic regimen they had been given. The first group comprised all patients treated with benzylpenicillin (*n* = 212, 44%), the second group all patients treated with a WSBL antibiotic (total *n* = 230, 48%; cefotaxim (*n* = 175), piperacillin-tazobactam (*n* = 20), cefuroxime (*n* = 16), imipenem-cilastatin (*n* = 8), meropenem (*n* = 8), ampicillin (*n* = 2), ceftazidime (*n* = 1)), and the third group those who received alternative treatment options (*n* = 39, 8%). A comparison of clinical characteristics between the three groups is presented in Appendix Table 8. Concomitant empirical therapy with another agent active against *H. influenzae* was administered to 13 patients (6%) in the benzylpenicillin group (fluoroquinolone (*n* = 3), doxycycline (*n* = 1), aminoglycoside (*n* = 9)) and to 17 patients (7%) in the wide spectrum beta-lactam group (fluoroquinolone (*n* = 8), doxycycline (*n* = 3), aminoglycoside (*n* = 6)). Thus, 199 patients received benzylpenicillin monotherapy and 213 patients received WSBL monotherapy and were included in the multivariate analysis and formed the basis for propensity score matching (Fig. 1).

### Clinical outcome in the two treatment groups according to uni- and multivariate logistic regression analysis

In the group receiving benzylpenicillin as empirical monotherapy, 11 patients (5%) died within 30 days after cultures were taken. In the group receiving WSBL monotherapy, 28 patients (13%) died. There was a significant difference between the two groups in terms of CCI, proportion of patients receiving ICU care, proportion of patients with bacteremia, and sepsis severity. Age, sex, maximal level of CRP, the proportion of immunosuppression, antimicrobial susceptibility to beta-lactam antibiotics, and CRB-65 score did not significantly differ between the two groups (Table 1).

**Table 1:** Descriptive characteristics of the non-adjusted final cohort on which the logistic regressions are performed, including all individuals receiving empirical monotherapy with benzylpenicillin (PcG) or a wide-spectrum beta-lactam (WSBL). Significant *p* values are in italics

Covariate		PcG, *n* = 199	WSBL^a^, *n* = 213	*P*	Missing values (*n*)
Age, *n* (%)				0.50	–
	0–40 years	12 (6.0)	11 (5.2)		
	40–60 years	11 (5.5)	20 (9.4)		
	60–80 years	103 (51.8)	104 (48.8)		
	> 80 years	73 (36.7)	78 (36.6)		
Sex, *n* (%)	Female	112 (56.3)	128 (60.1)	0.43	–
Maximum CRP, median (IQR)		250 (173–322)	241 (172–311)	0.53	4
ICU care, *n* (%)		3 (1.5)	12 (5.7)	*0.024*	2
CCI category (age not included), *n* (%)				*0.001*	4
	0–1	82 (41.6)	69 (32.7)		
	2–3	87 (44.2)	84 (39.8)		
	4–5	24 (12.2)	35 (16.6)		
	> 5	4 (2.0)	23 (10.9)		
Bacteremia, *n* (%)		48 (24.1)	72 (33.8)	*0.031*	–
Immune suppression, *n* (%)		10 (5.0)	13 (6.1)	0.63	4
Sepsis severity, *n* (%)				*0.042*	8
	no SIRS	21 (10.6)	18 (8.7)		
	sepsis	162 (81.8)	158 (76.7)		
	severe sepsis	15 (7.6)	24 (11.7)		
	septic shock	–	6 (2.9)		
CRB-65, *n* (%)				0.12	108
	0–1	112 (72.2)	92 (61.7)		
	2	35 (22.6)	43 (28.9)		
	3–4	8 (5.2)	14 (9.4)		
**Potential effect modifiers**					
Beta-lactamase, *n* (%)		29 (14.6)	30 (14.2)	0.92	2
rPBP3, *n* (%)		53 (26.6)	40 (19.0)	0.064	2

^a^i.v. WSBL antibiotics (monotherapy only): cefotaxim (*n* = 162), piperacillin-tazobactam (*n* = 18), cefuroxime (*n* = 16), imipenem-cilastatin (*n* = 7), meropenem (*n* = 7), ampicillin (*n* = 1), ceftazidime (*n* = 1)

To compare all-cause 30-day mortality between the group treated with benzylpenicillin and the group treated with WSBL, uni- and multivariate logistic regression was performed (Table 2). In the univariate analyses, a significantly increased 30-day mortality was seen in the group treated with WSBL compared to the benzylpenicillin group. Increasing age, CCI score, sepsis severity, and CRB-65 score were also associated with higher 30-day mortality. In the fitted multivariate model, adjusted for age, sepsis severity, and CCI-score, antibiotic treatment with WSBL was no longer significantly associated with increased mortality (OR 2.03, 95%CI 0.91–4.50, *p* = 0.082).

**Table 2:** Univariate and multivariate logistic regressions with 30-day mortality as outcome. Significant *p* values in the univariate regressions are in italics

Thirty-day mortality (*n* = 410) Events = 39		Univariate OR (95% CI)	*p*	Multivariate adjusted OR (95% CI), *n* = 399	*p*	Missing values (*n*)
WSBL vs PcG (ref) empirical monotherapy		2.59 (1.25–5.35)	*0.010*	2.03 (0.91–4.50)	0.082	–
Age, continuous^a^		1.05 (1.02–1.08)	*0.003*	1.06 (1.03–1.10)	0.001	–
Sex, female vs male (ref)		0.73 (0.38–1.42)	0.35			–
Maximum CRP		1.00 (1.00–1.01)	0.21			4
ICU care		1.48 (0.32–6.81)	0.62			2
CCI category (age not included)						4
	0–1	ref. cat	–	ref. cat	–	
	2–3	1.53 (0.65–3.60)	0.33	0.94 (0.37–2.39)	0.89	
	4–5	2.17 (0.77–6.12)	0.15	1.06 (0.34–3.36)	0.92	
	> 5	6.64 (2.29–19.3)	*< 0.001*	5.44 (1.69–17.5)	0.004	
Bacteremia		1.79 (0.91–3.51)	0.093			–
Immune suppression		1.46 (0.41–5.16)	0.56			4
Sepsis severity						8
	no	ref. cat	–	ref. cat	–	
	sepsis	1.53 (0.35–6.73)	0.57	1.40 (0.30–6.42)	0.67	
	severe sepsis	5.4 (1.08–26.9)	*0.04*	5.21 (0.96–28.3)	0.056	
	septic shock	18 (2.1–153)	*0.008*	28.1 (2.49–316)	0.007	
CRB-65^b^						108
	0-1	ref. cat	–			
	2	3.5 (1.54–7.96)	*0.003*			
	3–4	6 (1.99–18.1)	*0.001*			

^a^Used as a continuous variable due to no events in the youngest age group

^b^Not used in the multivariate analysis due to the number of missing values

We also compared mortality in patients with only *H. influenzae* bacteremia treated with either benzylpenicillin or other intravenous beta-lactams in a multivariate logistic regression model (Appendix Table 9). Thirty-day mortality was higher in the WSBL group, bordering on statistical significance (age- and CCI-adjusted OR 4.86, 95% CI of 0.98–24, *p* = 0.054), compared to the benzylpenicillin group.

There was no significant association between empirical antibiotic treatment and 30-day readmission rates. Increasing age and CCI were both significantly associated with the risk of readmission in the multivariate regression model (Table 3).

**Table 3:** Univariate and multivariate logistic regressions with 30-day readmission as outcome. Significant *p* values in the univariate regressions are in italics

Thirty-day readmission (*n* = 369) Events = 63		Univariate OR (95% CI)	*p*	Multivariate adjusted OR (95%CI), *n* = 369	*p*	Missing values (*n*)
WSBL vs PcG (ref) empirical monotherapy		1.23 (0.72–2.13)	0.45	1.16 (0.66–2.05)	0.61	–
Age, continuous^a^		1.04 (1.01–1.06)	*0.003*	1.03 (1.01–1.06)	0.013	–
Sex, female vs male (ref)		0.95 (0.55–1.64)	0.84			–
Maximum CRP		1.00 (0.19–4.05)	0.87			2
ICU care		0.88 (0.32–6.81)	0.62			1
CCI category (age not included)						–
	0–1	ref. cat	–	ref. cat	–	
	2–3	2.27 (1.14–4.55)	*0.020*	1.80 (0.88–3.68)	0.11	
	4–5	2.84 (1.20–6.72)	*0.018*	2.23 (0.92–5.39)	0.075	
	> 5	4.73 (1.62–13.8)	*0.005*	3.98 (1.32–12.0)	0.014	
Bacteremia		1.44 (0.81–2.56)	0.21			–
Immune suppression		1.15 (0.37–3.55)	0.81			–
Sepsis severity						12
	no SIRS	ref. cat	–			
	sepsis	1.26 (0.47–3.40)	0.65			
	severe sepsis	2.56 (0.73–8.93)	0.14			
	septic shock	no events	–			
CRB-65^b^						93
	0-1	ref. cat	–			
	2	1.38 (0.67–2.84)	0.39			
	3–4	3.18 (1.09–9.28)	*0.035*			

^a^Used as a continuous variable due to no events in the youngest age group

^b^Not used in the multivariate analysis due to the number of missing values

In contrast to 30-day mortality and 30-day readmission rate, the proportion of cases with early clinical response (in which data could be obtained) was higher in the WSBL group (*n* = 170, 84%) compared with the benzylpenicillin group (*n* = 153, 81%). This difference was statistically significant in a multivariate regression model after adjustment for age, bacteremia, CCI, and sepsis severity (OR 2.28, 95% CI 1.21–4.31, *p* = 0.011) (Table 4).

**Table 4:** Univariate and multivariate logistic regressions with early clinical response as outcome. Significant *p* values in the univariate regressions are in italics

Early clinical response (*n* = 392) (events = 323)		Univariate OR (95%CI)	*p*	Multivariate, adjusted OR (95%CI), *n* = 385	*p*	Missing values (*n*)
WSBL vs PcG (ref) empirical monotherapy		1.28 (0.76–2.16)	0.35	2.28 (1.21–4.31)	0.011	–
Age, continuous^a^		0.98 (0.96–1.00)	*0.031*	0.97 (0.95–0.99)	0.012	–
Sex, female vs male (ref)		1.02 (0.60–1.73)	0.93			–
Maximum CRP		0.99 (0.99–1.00)	*< 0.001*			2
ICU care		0.17 (0.05–0.52)	*0.002*			–
CCI category (age not included)						–
	0–1	ref. cat	–	ref cat	–	
	2–3	1.33 (0.73–2.45)	0.35	2.72 (1.30–5.70)	0.008	
	4–5	0.97 (0.44–2.11)	0.93	1.33 (0.54–3.27)	0.532	
	> 5	0.54 (0.21–1.36)	0.19	0.47 (0.16–1.37)	0.17	
Bacteremia		0.28 (0.16–0.49)	*< 0.001*	0.37 (0.20–0.69)	0.002	–
Immune suppression		2.09 (0.48–9.20)	0.33			–
Sepsis severity						7
	no SIRS	ref. cat	–	ref. cat		
	sepsis	0.31 (0.072–1.34)	0.12	0.27 (0.06–1.21)	0.087	
	severe sepsis	0.06 (0.013–0.30)	*0.001*	0.05 (0.010–0.27)	**<** 0.001	
	septic shock	0.01 (0.001–0.18)	*0.001*	0.009 (0.001–0.15)	0.001	
CRB-65^b^						98
	0-1	ref. cat	–			
	2	0.75 (0.37–1.51)	0.41			
	3–4	0.17 (0.06–0.43)	*< 0.001*			

^a^Used as a continuous variable due to no non-events in the youngest age group

^b^Not used in the multivariate analysis due to the number of missing values

### Clinical outcomes in the two treatment groups in the propensity score-matched cohort

To further adjust for potential confounders between the two treatment groups, a propensity score-matched cohort was constructed. The matched cohort consisted of 151 individuals treated with empirical benzylpenicillin monotherapy and 151 individuals receiving empirical WSBL treatment. In this matched cohort, 9 patients (6%) died within 30 days in the benzylpenicillin group and 18 patients (12%) died in the WSBL group (Appendix Table 10). Table 5 shows the balance in covariates between the two groups. The propensity score matching resulted in the omission of the most severely ill patients in the WSBL group, which is reasonable considering that these patients are not recommended empirical benzylpenicillin treatment.

**Table 5:** Descriptive characteristics for the 8 covariates matched for in the propensity-matched cohort, based on individuals receiving empirical monotherapy with benzylpenicillin (PcG) or a wide-spectrum beta-lactam (WSBL), using a caliper of 0.2 (*n* = 302)

Covariate		PcG, *n* = 151	WSBL, *n* = 151	*p*	Missing values (*n*)
Age, *n* (%)				0.97	–
	0–40 years	8 (5.3)	7 (4.6)		
	40–60 years	11 (7.3)	13 (8.6)		
	60–80 years	74 (49.0)	74 (49.0)		
	> 80 years	58 (38.4)	57 (37.8)		
Sex, *n* (%)	Female	95 (62.9)	91 (60.3)	0.64	–
Maximum CRP, median (IQR)		223 (142–306)	226 (167–304)	0.74	–
ICU care, *n* (%)		3 (2.0)	2 (1.3)	0.65	–
CCI category (age not included), *n* (%)				0.93	–
	0–1	53 (35.1)	58 (38.4)		
	2–3	71 (47.0)	68 (45.0)		
	4–5	23 (15.2)	22 (14.6)		
	> 5	4 (2.7)	3 (2.0)		
Bacteremia, *n* (%)		38 (25.1)	41 (27.2)	0.69	–
Immune suppression, *n* (%)		8 (5.3)	9 (6.0)	0.80	–
Sepsis severity, *n* (%)				0.63	–
	no SIRS	13 (8.6)	17 (11.3)		
	sepsis	126 (83.4)	123 (81.5)		
	severe sepsis	12 (8.0)	10 (6.6)		
	septic shock	–	1 (0.7)		
CRB-65^a^, *n* (%)				0.96	79
	0–1	82 (70.0)	73 (68.9)		
	2	28 (23.9)	27 (25.5)		
	3–4	7 (6.0)	6 (5.7)		

^a^Not used for matching due to the number of missing values

Thirty-day mortality was still higher, but not significantly higher in the WSBL group when comparing the matched groups both in a full cohort logistic regression model (OR 2.14, 95% CI 0.93–4.92, *p* = 0.075) and in a conditional regression model (OR 1.89, 95% CI 0.84–4.23, *p* = 0.12). There were still no significant differences in 30-day readmission rates (Appendix Table 10). Early clinical response rates remained significantly higher in the WSBL group both by full cohort logistic regression (OR 2.14, 95% CI 1.07–4.27, *p* = 0.031) and by conditional logistic regression (OR 2.5, 95% CI 1.20–5.21, *p* = 0.014).

### Effect modification

In order to evaluate any potential effect modification by beta-lactamase production and rPBP3, the odds ratios of the stratified outcomes per treatment group and resistance mechanism in the propensity-matched cohort were compared (Appendix Tables 11and 12). The presence of a beta-lactamase was a significant effect modifier of the association between treatment group and early clinical response (the interaction term for beta-lactamase × treatment group (benzylpenicillin as reference): β = 3.12, 95% CI 0.82–5.43, *p* = 0.008), explaining a substantial portion of the difference in early clinical response. This was not the case for isolates with rPBP3 (the interaction term for rPBP3 × treatment group (benzylpenicillin as reference): β = 0.74, 95% CI − 1.05–2.54, *p* = 0.42). Neither the associations between all cause 30-day mortality nor between 30-day readmission rates and treatment group were significantly modified by the presence of either resistance mechanism.

## Discussion

In the present study, the all-cause 30-day mortality of severe lower respiratory tract infections caused by *H. influenzae* was 9%. In an analysis adjusted for potential confounders, empirical monotherapy with benzylpenicillin was not significantly associated with increased risk of mortality or readmission. Early clinical response rates were high in both treatment groups, but significantly higher in the group receiving empirical monotherapy with a WSBL in an adjusted analysis, a difference largely explained by effect modification of benzylpenicillin treatment by beta-lactamase-producing strains.

Previous studies have reported mortality rates between 8 and 22% for *Haemophilus* bacteremia, results that are in good agreement with our findings of 12% in the bacteremia cohort [4, 5, 16]. Data on fatality rates of in-patients with pneumonia is scarce, with one recent study reporting a 30-day case fatality ratio of 2% [24]. This is lower than what we found in the non-bacteremia group (7%). The lower overall mortality in our study (9%) supports the notion that lower respiratory tract infections with *H. influenzae* are generally associated with less severe presentation compared with *S. pneumoniae* [1].

To the best of our knowledge, only one previous report has compared treatment outcome of benzylpenicillin in severe infections by *H. influenzae* [16]. That study was done in a retrospective cohort of bacteremia cases with various foci of infection, in Copenhagen, Denmark. The authors found a significantly increased 30-day mortality when using benzylpenicillin as a definitive treatment for bacteremia. As for empirical treatment, the results were not significant, but there was a trend towards higher mortality in the benzylpenicillin group (*p* = 0.06). This study also had a higher overall case fatality rate, reaching 22%. These results contrast our findings, where no such difference in outcome could be shown between treatment groups, neither in the bacteremia cohort nor in the overall study cohort. Since our study population only comprised respiratory tract infections, and empirical, as opposed to definite, treatment with benzylpenicillin, the results are not fully comparable.

In another recent, propensity score-matched study, benzylpenicillin and phenoxymethylpenicillin were compared with WSBL as empirical treatment of pneumonia with CRB-65 score ≤ 2 regardless of etiological agent [25]. No significant difference in mortality was seen between the two groups.

In contrast to 30-day mortality, there was a significantly lower chance of early clinical response among patients treated with benzylpenicillin in our study. However, the early clinical response rate in the benzylpenicillin group was still above 80%, and beta-lactamase production was found to be a significant effect modifier. When comparing patients infected with non-beta-lactamase-producing isolates in the propensity-matched cohort, early clinical response rates were 87% in the benzylpenicillin group compared to 89% in the WSBL group (Appendix Table 11).

The strength of this study includes its substantial size of an unselected, population-based homogenous cohort of cases with *H. influenzae* respiratory tract infection and a thorough analysis of data. The risk of an indication bias in a retrospective analysis of antibiotic treatment is always substantial. To counter this, the treatment groups were adjusted for known covariates that could confound the outcome association, including age, comorbidities, infection severity, and maximal level of CRP. However, the retrospective design of the study still entails a risk for residual confounding, supported by the counter-intuitive result that the mortality was higher in the wide-spectrum treatment group, and that this difference in outcome was bordering significance in multivariate analysis even after adjusting for confounders. On the other hand, this type of study would be very challenging to perform prospectively, as the causative agent in CAP is rarely known at the start of treatment. Another limitation of this study is its limited power due to the relatively low mortality rate. Given the number of patients in the two treatment groups (*n* = 199 for the benzylpenicillin group and *n* = 213 for the WSBL group), a defined α = 0.05 and β = 0.8, and a mortality of 14% in the WSBL group, this study has power to significantly detect an odds ratio of approximately 2, assuming a two-sided test of equality.

We have not performed a statistical comparison between high-dose (3 g t.i.d or higher) and low-dose (1 g t.i.d. or less) benzylpenicillin treatment. Monte Carlo simulations calculated by EUCAST suggest significantly lower target attainment rates for the latter regimen, which might lead to treatment failure [11]. Therefore, current Swedish guidelines advocate the use of high-dose benzylpenicillin in CAP treatment [9]. In our cohort, the fraction of patients receiving high-dose versus low-dose treatment were quite similar (55% (116/212) versus 44% (93/212), appendix Table 8). There was a trend towards fewer case fatalities in the high-dose group (*n* = 4 versus *n* = 7), but the number of events was too small to allow further statistical comparison.

Almost all rPBP3 isolates in Europe are still considered susceptible to third generation cephalosporins, whereas the susceptibility to aminopenicillins, and thus also to regular benzylpenicillin remains a matter of controversy [26]. In our study, the presence of rPBP3 isolates does not seem to have any detrimental effect on the clinical outcome in the benzylpenicillin group compared to patients treated with WSBL. Beta-lactamase production, in contrast to rPBP3, was a significant effect modifier, associated with a risk of reduced early clinical response in the benzylpenicillin group. This is intuitive, since third generation cephalosporins are generally stable to beta-lactamases expressed by *H. influenzae*, whereas benzylpenicillin is not [14]. Thus, beta-lactamase expression may well account for cases of treatment failure in this group. The proportion of beta-lactamase producing strains, however, has been stable over the past decades, whereas the increase in rPBP3 isolates has been worrisome [12, 13].

There has been debate on the optimal empirical treatment of respiratory tract infections and CAP caused by *H. influenzae*. Although definitive therapy with an agent with clinical breakpoints against *H. influenzae* always should be used following a positive culture, the present study suggests that empirical benzylpenicillin treatment is not associated with higher mortality in patients with mild to moderate lower respiratory tract infection caused by *H. influenzae.*

## Appendix

**Table 6:** Patients excluded from the initial cohort due to other foci of infection or other diagnosis

Patients excluded from bacteremia group due to other foci of infection	
Epiglottitis	*n* = 14
Meningitis	*n* = 11
Abdominal infection	*n* = 8
Urinary Tract Infection	*n* = 3
Arthritis	*n* = 3
Peritoneal infection	*n* = 2
Gynecological infection	*n* = 2
Soft tissue infection	*n* = 2
Aortitis	*n* = 1
Unknown focus of infection	*n* = 7
Incomplete medical records	*n* = 21
Total	*n* = 74
**Patients excluded from the sputum/nasopharyngeal sample group due to other diagnosis**	
Other respiratory tract pathogens	
Influenza virus	*n* = 11
*Streptococcus pneumoniae*	*n* = 6
Respiratory syncytial virus	*n* = 4
*Mycoplasma pneumoniae*	*n* = 3
β-hemolytic Streptococci	*n* = 3
*Legionella pneumonia*	*n* = 1
*Pneumocystis jirovecii*	*n* = 1
*Chlamydophila pneumoniae*	*n* = 1
Total	*n* = 30
Other focus of infection than the respiratory tract (w or w/o other pathogens)	
Urinary tract infection	*n* = 7
*Escherichia coli* bacteremia	*n* = 4
*Staphylococcus aureus* bacteremia	*n* = 3
*Streptococcus mitis* bacteremia	*n* = 2
*Pseudomonas aeruginosa* bacteremia	*n* = 1
Erysipelas	*n* = 1
*Clostridium difficile* enteritis	*n* = 1
Unknown focus of infection	*n* = 9
Total	*n* = 28
No signs of infection	*n* = 20
Total	*n* = 78

**Table 7:** Detailed description of the empirical antibiotic therapy given

Description of the empirical antibiotic treatment given:			
Empiric antibiotic treatment (*n*, %)	Group 1)	Benzylpenicillin	212 (44%)
		Total	212 (44%)
	Group 2)	Cefotaxime	175 (36%)
		Cefuroxime	16 (3%)
		Piperacillin-tazobactam	20 (4%)
		Imipenem-cilastatin	8 (2%)
		Meropenem	8 (2%)
		Ampicillin	2 (**<** 1%)
		Ceftazidime	1 (**<** 1%)
		Total	230 (48%)
	Group 3)	Doxycycline	12 (2%)
		Clindamycine	6 (1%)
		Amoxicillin	5 (1%)
		Erythromycine	3 (**<** 1%)
		Ciprofloxacine	2 (**<** 1%)
		Phenoximethyl penicillin	2 (**<** 1%)
		Trimetoprim /	
		sulfamethoxazole	2 (**<** 1%)
		Amoxicillin /	
		clavulanic acid	1 (**<** 1%)
		Cloxacillin	1 (**<** 1%)
		Levofloxacine	1 (< 1%)
		Roxithromycine	1 (**<** 1%)
		No antibiotic treatment	2 (**<** 1%)
		Missing data	1 (**<** 1%)
		Total	39 (8%)
Allergy to penicillin (*n*, %)		No	461 (96%)
		Yes	15 (3%)
		Missing data	5 (1%)
Concomitant antibiotic treatment active against			
H. influenzae in Benzylpenicillin group		None	199 (94%)
(*n*, % within group)			
		Fluoroquinolone	3 (1%)
		Doxycycline	1 (**<** 1%)
		Aminoglycoside (1 dose)	9 (4%)
		Total	212 (100%)
Concomitant antibiotic treatment active against			
H. influenzae in wide-spectrum beta-lactam group		None	213 (93%)
(*n*, % within group)			
		Fluoroquinolone	8 (4%)
		Doxycycline	3 (1%)
		Aminoglycoside (1 dose)	6 (3%)
		Total	230 (100%)
Concomitant antibiotic treatment active against			
H. influenzae in the miscellaneous group		None	36 (92%)
(*n*, % within group)			
		Fluoroquinolone	1 (2%)
		Doxycycline	1 (2%)
		Aminoglycoside (1 dose)	1 (2%)
		Total	39 (100%)
Dosage of benzylpenicillin		1 g t.i.d.	84 (40%)
(*n*, % within total group)		3 g t.i.d.	104 (49%)
		1 g b.i.d.	9 (4%)
		2 g t.i.d.	11 (6%)
		1 g q.i.d.	1 (**<** 1%)
		Missing data	3 (1%)
		Total	212 (100%)
30-day mortality (no of casualties, % within dosage group)			
in the benzylpenicillin group sorted by dosage			
		1 g t.i.d.	5 (6%)
		3 g t.i.d.	4 (4%)
		1 g b.i.d.	2 (22%)
		Other dosages	0

**Table 8:** Descriptive characteristics of the crude cohort, including all individuals in the study, stratified on treatment group. Significant differences are italics

Potential confounders		PcG, *n* = 212	WSBL^a^, *n* = 230	Other/no antibiotic *n* = 39	*p*	Missing values (*n*)
Age *n* (%)					0.13	–
	0–40 years	12 (5.7)	11 (4.8)	6 (15.4)		
	40–60 years	13 (6.1)	20 (8,7)	5 (12.8)		
	60–80 years	110 (51.9)	115 (50.0)	18 (46.2)		
	> 80 years	77 (36.3)	84 (36.5)	10 (25.6)		
Sex *n* (%)	Female	118 (55.7)	137 (59.6)	26 (66.7)	0.39	–
MaxCRP (median)		251	242	127	*< 0.001*	4
ICU care *n* (%)		5 (2.4)	15 (5.7)	1	0.082	2
CCIcat (unadjusted for age) *n* (%)					*< 0.001*	4
	0–1	89 (42.4)	75 (32.9)	21 (53.9)		
	2–3	90 (42.9)	90 (39.5)	11 (28.2)		
	4–5	27 (12.9)	38 (16.7)	7 (18.0)		
	> 5	4 (1.9)	25 (11.0)	–		
Bacteremia *n* (%)		53 (25.0)	78 (33.9)	9 (23.1)	0.082	–
Immune suppression *n* (%)		11 (5.2)	14 (6.1)	–	0.28	4
Sepsis severity *n* (%)					0.12	11
	no SIRS	22 (10.4)	18 (8.1)	5 (13.9)		
	sepsis	170 (80.6)	172 (77.1)	28 (77.8)		
	severe sepsis	19 (9.0)	25 (11.2)	2 (5.6)		
	septic shock	–	8 (3.6)	1 (2.8)		
CRB65 *n* (%)					0.26	123
	0–1	119 (71.7)	100 (60.6)	12 (70.6)		
	2	36 (21.7)	48 (29.1)	3 (17.7)		
	3–4	11 (6.6)	17 (10.3)	2 (11.8)		
Potential effect modifiers						
Beta-lactamase *n* (%)		32 (15.1)	31 (13.6)	2 (5.1)	0.25	2
rPBP3 *n* (%)		56 (26.4)	44 (19.3)	8 (20.5)	0.19	2
Low dose Benzyl-penicillin^b^ *n* (%)		93 (45.6)	–			3

^a^Wide spectrum i.v. beta lactam antibiotics: cefotaxim (*n* = 175), piperacillin-tazobactam (*n* = 20), cefuroxime (*n* = 16), imipenem-cilastatin (*n* = 8), meropenem (*n* = 8), ampicillin (*n* = 2), ceftazidime (*n* = 1)

^b^1 g t.i.d. or lower

**Table 9:** Univariate and multivariate logistic regressions with outcome 30-day mortality in only bacteremic isolates receiving empirical monotherapy with an i.v. beta-lactam. Significant *p* values in the univariate regressions are in italics

Thirty-day mortality, (*n* = 120) Events = 16		Univariate odds ratio (95%CI)	*P*	Multivariate adjusted OR (95%CI), *n* = 116	*p*	Missing values (*n*)
WSBL vs PcG (ref) empirical monotherapy		5.55 (1.20–25.7)	*0.028*	4.86 (0.98–24)	0.054	–
Age, continuous^a^		1.05 (1.00–1.10)	*0.004*	1.06 (1.01–1.12)	0.025	–
Sex, female vs male (ref)		0.68 (0.24–1.95)	0.47			–
Maximum CRP		1.00 (1.00–1.01)	0.35			4
ICU care		no events	–			2
CCI category (age not included)						4
	0–1	ref. cat	–	ref cat	–	
	2–3	1.09 (0.27–4.33)	0.91	0.65 (0.15–2.83)	0.57	
	4–5	3.33 (0.63–17.6)	0.16	1.65 (0.26–10.3)	0.59	
	> 5	8.0 (1.51–42.4)	*0.015*	4.59 (0.74–28.6)	0.10	
Immune suppression		no events	–			2
Sepsis severity						6
	No	ref. cat	–			
	sepsis	0.72 (0.08–6.86)	0.78			
	severe sepsis	0.71 (0.06–8.40)	0.79			
	septic shock	3.33 (0.20–54)	0.40			
CRB65^b^						47
	0-1	ref. cat	–			
	2	1.75 (0.39–7.8)	0.46			
	3–4	3.75 (0.68–21)	0.13			

^a^Used as a continuous variable due to no events in the youngest age group

^b^Not used in the multivariate analysis due to the number of missing values

**Table 10:** Outcomes stratified by treatment group in the propensity-matched cohort

No	*n* (%)		logistic regression, OR (95%CI)			conditional logistic regression, OR (95%CI)		
	Benzyl-penicillin	WSBL	Benzyl-penicillin	WSBL	*p* value	Benzyl-penicillin	WSBL	*p* value
30-day all cause mortality, (*n* = 300)	9/150 (6.0%)	18/150 (12.0%)	1 (ref)	2.14 (0.93–4.92)	0.075	1 (ref)	1.89 (0.84–4.23)	0.12
30-day all cause hospital readmission, (*n* = 275)	24/143 (16.8%)	21/132 (15.9%)	1 (ref)	0.94 (0.49–1.78)	0.85	1 (ref)	0.89 (0.46–1.72	0.74
Early clinical response at day 4 (*n* = 292)	119/146 (81.5%)	132/146 (90.4%)	1 (ref)	2.14 (1.07–4.27)	0.031	1 (ref)	2.5 (1.20–5.21)	0.014

**Table 11:** Potential effect modification of the treatment group by betalactamase production on the respective outcomes in the propensity-matched cohort, balanced by covariates earlier described

Thirty-day all-cause mortality (*n* = 300)	Benzylpenicillin treatment		Wide-spectrum betalactamase treatment		OR within STRATA of beta-lactamase presence
	*n* with outcome (%)	OR (95% CI), *p*		OR (95% CI), *p*	
Isolates without betalactamase	8/129 (6.2%)	1 (ref)	16/125 (12.8%)	2.22 (0.91–5.39), *p* = 0.078	2.22 (0.91–5.39), p = 0.078
Isolates with beta-lactamase	1/21 (4.7%)	0.76 (0.090–6.38), *p* = 0.80	2/25 (8.0%)	1.32 (0.26–6.60), *p* = 0.74	1.74 (0.15–20.6), *p* = 0.66
30-day all-cause readmission (*n* = 275)					
Isolates without betalactamase	21/124 (16.9%)	1 (ref)	19/108 (17.6%)	1.05 (0.53–2.07), *p* = 0.90	1.05 (0.53–2.07), p = 0.90
Isolates with beta-lactamase	3/19 (15.8%)	0.92 (0.25–3.44), p = 0.90	2/24 (8.3%)	0.45 (0.097–2.04), *p* = 0.30	0.48 (0.072–3.25), *p* = 0.46
Early clinical response on day 4 (*n* = 292)					
Isolates without betalactamase	109/125 (87.2%)	1 (ref)	107/120 (89.2%)	1.21 (0.55–2.63), *p* = 0.63	1.21 (0.55–2.63), p = 0.63
Isolates with beta-lactamase	10/21 (47.6%)	0.13 (0.05–0.36), *p* < 0.001	25/26 (96.1%)	3.67 (0.46–29.0), *p* = 0.22	27.5 (3.13–242), *p* = 0.003

Interaction term for betalactamase × treatment group (Benzyl-pc as ref) for 30-day mortality: β = − 0.24 (− 2.97–2.38), *p* = 0.86

Interaction term for betalactamase × treatment group (Benzyl-pc as ref) for 30-day readmission: β = − 0.77 (− 2.79–1.25), *p* = 0.46

Interaction term for betalactamase × treatment group (Benzyl-pc as ref) for early clinical response: β = 3.12 (0.82–5.43), *p* = 0.008

**Table 12:** Potential effect modification of the treatment group by rPBP3 on the respective outcomes in the propensity-matched cohort, balanced by covariates earlier described

Thirty-day all-cause mortality (*n* = 300)	Benzylpenicillin treatment		Wide-spectrum betalactamase treatment		OR within STRATA of rPBP3
	*n* with outcome (%)	OR (95% CI), *p*		OR (95% CI), *p*	
Isolates without rPBP3	4/110 (3.6%)	1 (ref)	13/121 (10.7%)	3.19 (1.01–10.1), *p* = 0.048	3.19 (1.01–10.1), *p* = 0.048
Isolates with rPBP3	5/40 (12.5%)	3.79 (0.96–14.9), *p* = 0.06	5/29 (17.2%)	5.52 (1.38–22.1), *p* = 0.016	1.46 (0.38–5.6), *p* = 0.58
30-day all-cause readmission (*n* = 275)					
Isolates without rPBP3	17/106 (16.0%)	1 (ref)	19/106 (17.9%)	1.14 (0.56–2.34), *p* = 0.72	1.14 (0.56–2.34), *p* = 0.72
Isolates with rPBP3	7/37 (18.9%)	1.22 (0.46–3.23), *p* = 0.69	2/26 (7.7%)	0.44 (0.094–2.02), *p* = 0.29	0.35 (0.068–1.88), *p* = 0.22
Early clinical response on day 4 (*n* = 292)					
Isolates without rPBP3	87/105 (82.9%)	1 (ref)	105/117 (89.7%)	1.81 (0.83–3.96), *p* = 0.14	1.21 (0.55–2.63), *p* = 0.63
Isolates with rPBP3	32/41 (78.0%)	0.74 (0.30–1.80), *p* = 0.50	27/29 (93.1%)	2.79 (0.61–12.8), *p* = 0.19	3.80 (0.75–19.1), *p* = 0.11

Interaction term for rPBP3 × treatment group (Benzyl-pc as ref) for 30-day mortality: β = − 0.78 (− 2.55–0.99), *p* = 0.39

Interaction term for rPBP3 × treatment group (Benzyl-pc as ref) for 30-day readmission: β = − 1.16 (− 2.97–0.65), *p* = 0.21

Interaction term for rPBP3 × treatment group (Benzyl-pc as ref) for early clinical response: β = 0.74 (− 1.05–2.54), *p* = 0.42
